# Exploring Pharmacokinetic interactions between SHR8554, a µ-opioid receptor biased agonist, and Itraconazole in healthy Chinese subjects

**DOI:** 10.1038/s41598-025-98697-3

**Published:** 2025-07-02

**Authors:** Lei Huang, Hao Jiang, Yuanyuan Huang, Juan Li

**Affiliations:** 1 Phase I Clinical Trials Unit, Affiliated Hospital of Medical School, Nanjing Drum Tower Hospital, Nanjing University, No. 321 Zhongshan Road, Gulou District, Nanjing, 210008 China; 2 Jiangsu Hengrui Medicine Co., Ltd., Lianyungang, China; 3 Clinical Pharmacology Department, Jiangsu Hengrui Medicine Co., Ltd., No. 7 Kunlun Mountain Road, Lianyungang Economic and Technological Development Zone, Lianyungang, 222000 China

**Keywords:** Biased agonist, CYP3A4 inhibitors, Drug-drug interaction, Μ- opioid receptors, SHR8554, Drug safety, Pharmacokinetics

## Abstract

SHR8554 is a biased agonist for µ-opioid receptors, which was under investigation as an alternative therapy for clinical pain. This open, fixed-sequence study evaluated its pharmacokinetic (PK) interaction with itraconazole, a strong CYP3A4 inhibitor. Subjects (*n* = 16) were given SHR8554 (1 mg) intravenously on Day 1 and Day 9, and itraconazole capsules (200 mg) twice a day orally from Day 4 to Day 10. Liquid chromatography tandem mass spectrometry was applied for plasma concentration of SHR8554. After single dose of SHR8554, the C_max_ and T_max_ were 16.69 ± 3.48 ng/mL and 0.16 h, which was quite close to that of combination dose of itraconazole (C_max_ 16.58 ± 8.79 ng/mL, T_max_ 0.22 h). AUC_0−∞_ under monotherapy (18.37 ± 3.61 ng∙h/mL) and combinated therapy (19.91 ± 3.59 ng∙h/mL) were also approximate to each other. The main PK parameters were almost consistent between single dose of SHR8554 period and itraconazole combined period with the geometric mean ratios (90%CI) basically within 80–125%. Treatment-emergent adverse events occurred much less in single dose period than combination period (75.0% vs. 93.8%). Therefore, when co-administered with CYP3A4 inhibitors, SHR8554 maintained its distinctive PK profile while the subjects experienced an increase in symptoms associated with opioid receptor activation. The ClinicalTrials.gov identifier is NCT05928988 (03/07/2023).

**Supplementary Information:**

The online version contains supplementary material available at 10.1038/s41598-025-98697-3.

## Introduction

Postoperative pain is a common complication following surgeries, which should be alleviated to prevent other complications and promote the healing process^1^. Opioid agonists (opioids) are the reference standard for acute pain including postoperative pain^2^. There are four subtypes of opioid receptors including µ receptor (MOR), δ receptor (DOR), κ receptor (KOR) and the nociceptin peptide receptor (NOP)^3^. Morphine, oxycodone and fentanyl are the most effective analgesics that activate MOR, but limitations should not be overlooked due to their adverse events^4^. Therefore, novel opioids analgesics with less adverse side effects are driven to be developed.

SHR8554 injection is a novel analgesic developed by Jiangsu Hengrui Medicine Co., Ltd. which is a highly potent and selective agonist for the MOR, with relatively weak activation of the β-arrestin signaling pathway^5^. SHR8554 demonstrated promising characteristics in terms of its molecular structure and half-life. The compound was refined to investigate crucial pharmacophoric features, particularly the steric influence of its phenyl-rich ring. Structural optimization revealed that the S-configuration amine and S-configuration ethoxy groups contribute to strong MOR activation while maintaining low β-arrestin 2 signaling. This selective profile enhances its potency while minimizing undesirable pathway activation^6^. In a Phase III trial (NCT04766463/NCT05375305), SHR8554 at doses of 0.05 mg and 0.1 mg (administered as a 1 mg loading dose followed by patient-controlled analgesia (PCA) doses of 0.05 mg or 0.1 mg) demonstrated significant efficacy over placebo in alleviating moderate to severe acute pain following unilateral total knee replacement or knee ligament reconstruction. The injection was well-tolerated with a favorable safety profile, suggesting that SHR8554 could be a promising option for postoperative pain management^6^.

Additionally, it was proved that cytochromes P450 (CYP) enzymes including CYP2D6, CYP3A4 and CYP3A5 were the major metabolic enzymes of SHR8554. SHR8554 exhibited moderate inhibition of CYP2D6 (IC_50 NADPH_^+^ = 6.35 µM and IC_50 NADPH_^−^ = 5.39 µM), but it did not exhibit time-dependent inhibition. It also inhibited CYP3A4/5 in two different reactions. For the midazolam 1’-hydroxylation reaction, the IC_50 NADPH_^+^ value was 8.87 µM and the IC_50 NADPH_^−^ value was 17.2 µM. For the testosterone 6β-hydroxylation reaction, the IC_50 NADPH_^+^ value was 18.6 µM and the IC_50 NADPH_^−^ value was greater than 100 µM. The shift in IC_50_ values with and without NADPH for both CYP3A4/5 reactions was greater than 1.5-fold, indicating time-dependent inhibition. The inhibition of these CYP enzymes could result in decreased drug metabolism, which might cause high drug concentrations in plasma and related clinical toxicities^7^.

In this study, we explored the pharmacokinetics and tolerance of SHR8554 when combined with itraconazole ( a CYP3A4 inhibitor) to pave the way for clinical administration.

## Results

### Population

**Fig. 1:**
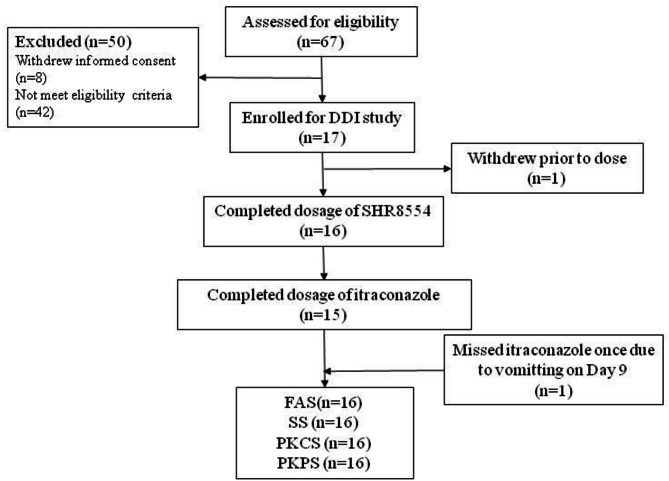
Subject assignment. *FAS* full analysis set, *SS* safety set, *PKCS* pharmacokinetic concentration set, *PKPS* pharmacokinetics parameter set.

Initially, 17 subjects were qualified for the study, screened out from 67 candidates (Fig. 1). One subject withdrew the informed consent before administration. Ultimately, 5 females and 11 males were included and their characteristics are detailed in Table 1.

**Table 1:** Demographics and baseline characteristics of all subjects.

Terms	Total (*N* = 16)
Age (years)	27.8 (6.83)
Gender, n (%)	
Male	11 (68.8)
Female	5 (31.3)
Nationality, n (%)	
Han	15 (93.8)
Others	1 (6.3)
Height (cm)	168.22 (11.231)
Weight (kg)	62.14 (11.219)
BMI (kg/m^2^)	21.83 (2.105)

Age, height, weight and BMI were presented in mean (SD).

*BMI* body mass index, *SD* standard deviation.

### PK results and DDI evaluation

**Fig. 2:**
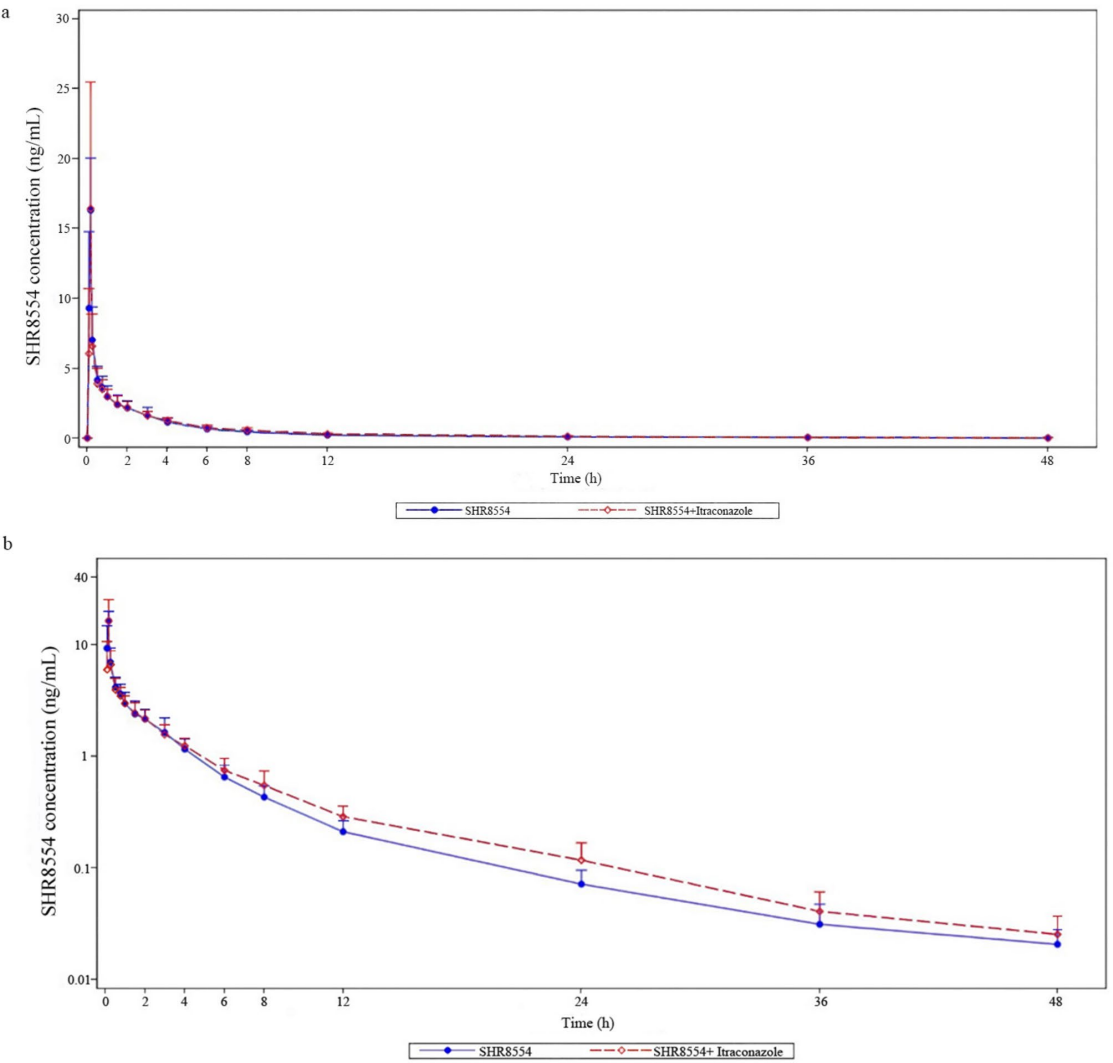
The linear (**a**) and semi-logarithmic (**b**) mean ± SD plasma concentration-time profiles of SHR8554 alone or combined with itraconazole. *SD* standard deviation.

The concentration – time (C-T) curves are presented in Fig. 2. After single dose of SHR8554, the C_max_ and T_max_ were 16.69 ± 3.48 ng/mL and 0.16 h, which was quite close to that of combination dose of itraconazole (C_max_ 16.58 ± 8.79 ng/mL, T_max_ 0.22 h). AUC_0 − t_ and AUC_0−∞_ were also silmilar to each other. The main PK characteristics were shown in Table 2. The 90% CIs of the geometric mean ratio (GMR) of the PK parameters were basically within 80–125%, except the lower boundary of 90% CI of GMR of C_max_ (0.64) was slightly lower than 0.8. T_max_ was analyzed by wilconxon paired rank sum test and showed no difference between single and combined dose group. (Tables 3 and 4)

**Table 2:** Main PK parameters of SHR8554 in plasma after a single intravenous pump infusion of 1 mg SHR8554 and combined administration with Itraconazole in healthy subjects.

Parameters (unit)	Groups	
	Monotherapy	Combination therapy
C_max_ (ng/mL)	16.69 ± 3.48 (20.9)	16.58 ± 8.79 (53.0)
T_max_* (h)	0.16 (0.0828, 0.1678)	0.22 (0.1636, 0.7489)
AUC_0–t_ (ng∙h/mL)	18.10 ± 3.60 (19.9)	19.58 ± 3.51 (17.9)
AUC_0−∞_ (ng∙h/mL)	18.37 ± 3.61 (19.7)	19.91 ± 3.59 (18.0)
AUC_0 − 4 h_ (ng∙h/mL)	11.42 ± 2.19 (19.2)	10.97 ± 2.25 (20.5)
AUC_0 − 6 h_ (ng∙h/mL)	13.23 ± 2.56 (19.4)	12.96 ± 2.45 (18.9)
AUC_0–12 h_ (ng∙h/mL)	15.59 ± 3.05 (19.6)	15.92 ± 2.89 (18.1)
AUC_0–24 h_ (ng∙h/mL)	17.26 ± 3.40 (19.7)	18.34 ± 3.26 (17.8)
T_1/2z_ (h)	9.28 ± 3.13 (33.7)	8.81 ± 2.06 (23.4)
V_z_ (L)	751.35 ± 315.01 (41.9)	653.26 ± 185.56 (28.4)
CL_z_ (L/h)	56.41 ± 11.00 (19.5)	51.77 ± 9.27 (17.9)
λ_z_ (/h)	0.09 ± 0.04 (46.7)	0.08 ± 0.02 (26.0)
MRT_0–t_ (h)	5.10 ± 0.95 (18.6)	6.23 ± 1.43 (23.0)
MRT_0−∞_(h)	5.95 ± 1.50 (25.2)	7.08 ± 1.96 (27.7)
AUC__%Extrap_(%)	1.51 ± 0.98 (65.0)	1.63 ± 1.00 (61.5)

*Median (minimum, maximum) was used for T_max_ and other parameters were described as Mean ± SD(%CV). C_max_, peak concentration; T_max_, peak time; λz, terminal elimination rate constant; T_1/2_, terminal elimination half-life; AUC_0 − t_, area under the curve from 0 to the last detectable time; AUC_0−∞_, area under the curve from 0 to infinite time; CLz, apparent clearance rate; Vz, apparent distribution volume; MRT_0 − t ,_ mean residence time from time 0 to the last detectable time; MRT_0−∞,_ mean residence time from time 0 to infinite time; AUC_%Extrap_, the extrapolated percentage of AUC_0−∞_.

**Table 3:** 90% CI results for SHR8554’s main PK parameters in the monotherapy group and in combination with Itraconazole.

Pharmacokinetic parameters	Groups (*n* = 16)	Geometric mean		GMR	
		Estimated value	95% CI	Estimated value	90% CI
C_max_ (ng/mL)	Combination therapy	14.05	(10.84, 18.20)	0.86	(0.64, 1.17)
	Monotherapy	16.30	(12.58, 21.12)		
AUC_0 − t_ (h*ng/mL)	Combination therapy	19.29	(17.44, 21.33)	1.09	(1.02, 1.15)
	Monotherapy	17.77	(16.07, 19.66)		
AUC_0−∞_ (h*ng/mL)	Combination therapy	19.61	(17.74, 21.67)	1.09	(1.02, 1.16)
	Monotherapy	18.05	(16.33, 19.94)		
t_1/2z_ (h)	Combination therapy	8.58	(7.22, 10.18)	0.98	(0.90, 1.08)
	Monotherapy	8.72	(7.34, 10.35)		
V_z_ (L)	Combination therapy	630.88	(527.11, 755.07)	0.91	(0.81, 1.01)
	Monotherapy	697.08	(582.42, 834.30)		
CL_z_ (L/h)	Combination therapy	51.00	(46.14, 56.36)	0.92	(0.87, 0.98)
	Monotherapy	55.41	(50.13, 61.24)		

CI: confidence interval; GMR, geometric mean ratio; C_max_, peak concentration; AUC_0 − t_, area under the curve from 0 to the last detectable time; AUC_0−∞_, area under the curve from 0 to infinite time; T_1/2_, terminal elimination half-life; CLz, apparent clearance rate; Vz, apparent distribution volume.

**Table 4:** T_max_ analysis of SHR8554 in the monotherapy group and in combination with Itraconazole.

Pharmacokinetic parameters	*P* value (combination vs. monotherapy)
T_max_ (h)	0.1085

T_max_, peak time.

### Safety and tolerability

In total, 57 cases (*n* = 16, 100%)of treatment emerged adverse events (TEAEs) were reported in this study (Table 5). The most frequent TEAEs were dizziness, nausea and vomiting both in the single dose phase and the combined dose phase. Compared to single dose, the combined dose phase had a higher ratio of TEAE ( 93.8% vs. 75%). During the combined phase, one vomiting event was moderate and intervened with ondansetron hydrochloride (4 mg) intravenously and all the other TEAEs were mild. Specially, there was no TEAE observed during D4 to D8, when itraconazole was taken without SHR8554. In the whole study, there were no severe adverse events or withdrawal events.

**Table 5:** Treatment emergent adverse events.

SOC/PT	SHR8554 (*N* = 16)		Itraconazole (*N* = 16)		SHR8554 + Itraconazole (*N* = 16)		Total (*N* = 16)	
	Case	*n* (%)	Case	*n* (%)	Case	*n* (%)	Case	*n* (%)
TEAE	21	12 (75.0)	0	0 (0.0)	36	15 ( 93.8)	57	16 (100.0)
Metabolic and nutritional disorders	0	0 (0.0)	0	0 (0.0)	2	1 (6.3)	2	1 (6.3)
Hypophosphatemia	0	0 (0.0)	0	0 (0.0)	1	1 (6.3)	1	1 (6.3)
Hypokalemia	0	0 (0.0)	0	0 (0.0)	1	1 (6.3)	1	1 (6.3)
Musculoskeletal and connective tissue disorders	0	0 (0.0)	0	0 (0.0)	1	1 (6.3)	1	1 (6.3)
Myalgia	0	0 (0.0)	0	0 (0.0)	1	1 (6.3)	1	1 (6.3)
Various examinations	3	2 (12.5)	0	0 (0.0)	5	4 (25.0)	8	5 (31.3)
Increased neutrophil count	1	1 (6.3)	0	0 (0.0)	1	1 (6.3)	2	2 (12.5)
Positive occult blood in urine	0	0 (0.0)	0	0 (0.0)	1	1 (6.3)	1	1 (6.3)
Positive leukocytes in urine	1	1 (6.3)	0	0 (0.0)	0	0 (0.0)	1	1 (6.3)
Prolonged QT interval on electrocardiogram	0	0 (0.0)	0	0 (0.0)	1	1 (6.3)	1	1 (6.3)
Elevated direct bilirubin	0	0 (0.0)	0	0 (0.0)	1	1 (6.3)	1	1 (6.3)
Elevated thyroid-stimulating hormone in blood	0	0 (0.0)	0	0 (0.0)	1	1 (6.3)	1	1 (6.3)
Elevated blood uric acid	1	1 (6.3)	0	0 (0.0)	0	0 (0.0)	1	1 (6.3)
Neurological disorders	11	10 (62.5)	0	0 (0.0)	17	15 (93.8)	28	15 (93.8)
Dizziness	10	10 (62.5)	0	0 (0.0)	15	14 (87.5)	25	15 (93.8)
Drowsiness	0	0 (0.0)	0	0 (0.0)	2	2 (12.5)	2	2 (12.5)
Headache	1	1 (6.3)	0	0 (0.0)	0	0 (0.0)	1	1 (6.3)
Cardiac organ disorders	1	1 (6.3)	0	0 (0.0)	0	0 (0.0)	1	1 (6.3)
Palpitations	1	1 (6.3)	0	0 (0.0)	0	0 (0.0)	1	1 (6.3)
Gastrointestinal system disorders	6	4 (25.0)	0	0 (0.0)	10	6 (37.5)	16	6 (37.5)
Nausea	4	4 (25.0)	0	0 (0.0)	6	5 (31.3)	10	6 (37.5)
Vomiting	2	2 (12.5)	0	0 (0.0)	4	4 (25.0)	6	4 (25.0)
Blood and lymphatic system disorders	0	0 (0.0)	0	0 (0.0)	1	1 (6.3)	1	1 (6.3)
Anemia	0	0 (0.0)	0	0 (0.0)	1	1 (6.3)	1	1 (6.3)

The time frame for collecting TEAEs: SHR8554: Day1 to Day3; Itraconazole: Day4 to Day8; SHR8554 + Itraconazole: Day9 to the end of the study. *TEAE* Treatment emergent adverse events.

## Discussion

It is well known that the activation of opioid receptors subsequently acts on two main pathways, the β-arrestin 2 or /and the G-protein pathways^8,9^. The concept of functional selectivity was established that activation of the G-protein pathway induced analgesic effect while that of the β-arrestin 2 pathway mainly triggered side effects^10^. The strategy of synthesizing biased agonists targeting the MOR could be traced back to the last century, when Bohn et al. found that inhibition of β-arrestin 2 function led to an enhancement in analgesic effectiveness of morphine^11^. The following studies gradually uncovered that the lack of β-arrestin 2 failed to develop antinociceptive tolerance and diminished the side effects including opiates-induced constipation and respiratory suppression^12–15^.

SHR8554 was such a novel analgesic that was designed as a biased agonist targeting the MOR and selectively activating the G-protein pathway^5^. According to previous phase I dose escalation study for SHR8554 (CTR20180587), the MTD was 2.5 mg (**Table ****S1**). In the phase III studies (NCT04766463/NCT05375305), the clinical dose of SHR8554 as a single agent included a 1 mg loading dose and a 0.05 mg effective dose administered via PCA pump^6^. Referring to drug interaction results with a similar drug, oliceridine^16^, in CYP2D6 poor metabolizers, continuous administration of 200 mg/day itraconazole for 5 days resulted in an approximately 80% increase in AUC_0 − inf_, with no significant impact on C_max_. Additionally, in CYP2D6 poor metabolizers, AUC_0 − inf_ of oliceridine increased approximately twofold compared to non-poor metabolizers. In preclinical studies, CYP2D6, CYP3A4 and CYP3A5 were proved to be the major metabolic enzymes of SHR8554. Thus, it was anticipated that the exposure level of 1 mg SHR8554 in healthy individuals, in the presence of CYP450 inhibitors, would be lower than the single maximum tolerated dose of 2.5 mg, making it safe and well-tolerated. Therefore, the dosage for SHR8554 injection in this study was determined to be 1 mg.

The previous study for SHR8554 (CTR20180587) also demonstrated that, after a single intravenous infusion of SHR8554 over 30 min in healthy subjects, plasma concentrations in the 0.75–3 mg dose groups reached their peak approximately at the end of the infusion (**Table ****S1**). The median T_max_ values across the dose groups were around 0.33–0.50 h, after which plasma concentrations declined following a biexponential function. There were no significant differences in distribution and elimination-related parameters across the dose groups. The elimination half-life (t_1/2_) ranged from 6.08 to 6.96 h, and the total clearance ranged from 45.12 to 55.90 L/h. Within the 0.75–3 mg dose range, SHR8554 injection administered via a single 30-minute intravenous infusion exhibited linear pharmacokinetics, as indicated by the C_max_ and AUC values. Additionally, the study revealed that there was no significant difference in the overall exposure of SHR8554 after administration with different infusion times (2–30 min) (**Table S2**).

Therefore, in this study, SHR8554 was also administered via intravenous infusion, with the blood concentration expected to peak around the time of infusion completion. Intensive blood sampling was conducted at key time points: 5 min after the infusion started, immediately after it ended, at 15 min and 30 min. To meet the requirement of sampling over at least three terminal elimination half-lives, and ensuring that the AUC_0 − t_/AUC_0−∞_ is greater than 80%, plasma concentrations were collected for at least 24 h post-dose. Additionally, considering that co-administration with CYP450 inhibitors may lead to increased exposure levels and slower metabolic elimination, plasma concentrations were collected up to 48 h after administration in this study.

The clinical recommended dosage for itraconazole is 100 mg or 200 mg, once daily or twice daily^17^. According to the guidance principles by the China Drug Evaluation (CDE), in clinical trials of DDI, the inducing drug should be studied at the maximum observable interaction dose under safe conditions. This includes using the maximum dose and the shortest dosing interval from the clinically recommended dosing regimen. Therefore, the dosage of itraconazole in this study was determined to be 200 mg, twice daily.

In multiple DDI studies, where 100–200 mg/day of itraconazole was administered with a 3-day lead-in, there was a consistent demonstration of robust inhibition of CYP3A, even though the 3-day period was insufficient to establish a steady-state of itraconazole^18–20^. Consequently, to achieve a more stable state with maximum CYP3A inhibition, itraconazole was administered for 5 days prior to SHR8554 in this study. Furthermore, to maintain adequate inhibition, the recommendation was to continue itraconazole for 4–5 half-lives of the substrate following co-administration^21^. Therefore, itraconazole was extended for an additional 2 days in this study, considering the short half-life (approximately 9 h) of SHR8554.

This study showed that a single 1 mg dose of SHR8554 resulted in an average C_max_ of 16.69 ng/mL and AUC_0 − t_ of 18.1 ng∙h/mL. The intravenous infusion lasted approximately 10 min, with plasma concentrations peaking near the end of infusion (Fig. 2). Thereafter, plasma levels declined in a biphasic exponential manner with a t_1/2_ of 9.28 h. After achieving relative steady-state of itraconazole, simultaneous administration of 1 mg SHR8554 resulted in an average C_max_ of 16.58 ng/mL and AUC_0 − t_ of 19.58 ng∙h/mL. Similarly, plasma concentrations peaked at approximately 13 min (0.22 h), coinciding with the end of the infusion. While the AUC_0 − t_ of SHR8554 under combination therapy showed a slight increase, the 90% CIs of GMR of the PK parameters were predominantly within 80–125%, indicating no significant difference when compared to the monotherapy of SHR8554 (Tables 3 and 4). This suggested that SHR8554 maintained its distinctive pharmacokinetic profile when co-administered with CYP3A4 inhibitors.

Additionally, the study results indicated that (**Table S3**), in both the monotherapy and combination therapy groups, the GMRs for SHR8554 C_max_, AUC_0 − t_, and AUC_0−∞_ between male and female subjects were all above 80%, suggesting no significant gender differences in SHR8554 exposure levels. However, the t_1/2_ for male subjects was approximately 22% shorter than for female subjects in both groups. The distribution volume (Vz) was similar between genders, while the total clearance (CLz) for male subjects was approximately 16% (monotherapy) to 24% (combination therapy) higher than for female subjects. Overall, no significant gender differences were observed in SHR8554 distribution and elimination parameters. Due to the limited sample size, the statistical inference regarding gender differences should be considered as preliminary.

Nevertheless, following the inhibition of CYP3A4 enzyme activity by itraconazole, there was an increasing trend in the incidence of TEAEs (93.8% vs. 75%) compared to when SHR8554 was administered alone. The primary heightened TEAEs following combination administration included dizziness, drowsiness, nausea, and vomiting, all associated with the activation of opioid receptors^22,23^. Fortunately, these TEAEs were generally tolerated. Among the 16 subjects who received the administration, a total of 4 subjects experienced vomiting on the day of co-administration of SHR8554 and itraconazole, with 1 subject not taking itraconazole due to vomiting later in the evening. Considering that this could potentially lead to a decrease in the bioavailability of itraconazole, thereby affecting the PK characteristics of SHR8554, a discussion and confirmation were carried out after data review. In the primary analysis, these 4 subjects were included, and a sensitivity analysis was conducted by excluding the SHR8554 blood concentration and its PK parameter data for these 4 subjects from both the monotherapy group and the combination therapy group. The conclusion of the drug interaction assessment after exclusion was consistent with the primary analysis conclusion. Based on the above findings, while the PK profile of SHR8554 did not exhibit significant changes after CYP3A4 inhibition, subjects remained at risk for heightened symptoms associated with opioid receptor activation.

## Subjects and methods

### Study information

This was a single-center, open, fixed-sequence drug-drug interactions (DDI) study, approved by the Chinese National Medical Products Administration(Approving number 2017L04801) and registered on the website of clinicaltrials.gov (NCT05928988, first posted date 03/07/2023). The protocol of this clinical trial was approved by the Medical Ethics Committee of Nanjing Drum Tower Hospital, the Affiliated Hospital of Nanjing University Medical School (Approving number 2021-541-02). All methods were performed in accordance with the relevant guidelines and regulations including but not limited to the Declaration of Helsinki and the Good Clinical Practice of China.

### Subjects

All subjects were willing to participate in this study and provided the informed consent with signature. Participants aged 18 to 45 years old and weighed over 45 kg (female) or 50 kg (male) with body mass index within 19 to 26 kg/m^2^ were enrolled. Any terms of health examination showed clinically significant or any history of medication or disease before administration were excluded.

### Study design

**Fig. 3:**
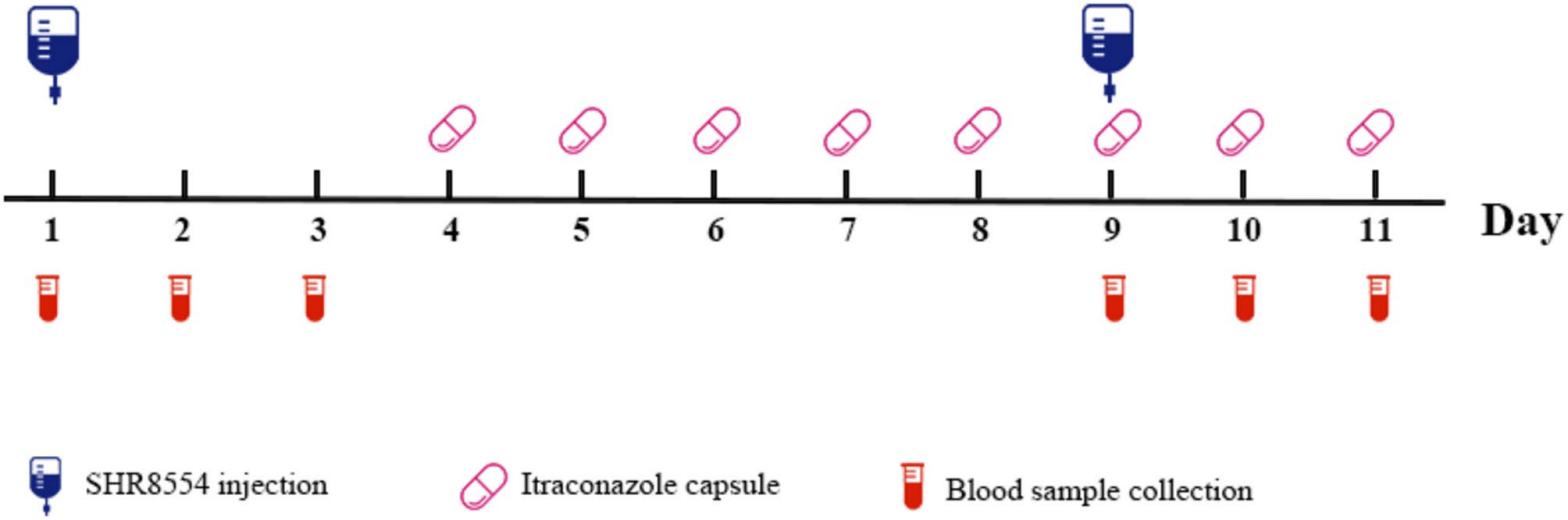
Drug–drug interactions study design.

Subjects were admitted to the phase I clinical trial unit on Day 0 and discharged on Day 11 (Fig. 3). On Day 1, SHR8554 (1 mg) was administered intravenously using an injection pump after breakfast. From Day 4 to Day 10, itraconazole (200 mg) was taken orally twice a day after breakfast and dinner. On the morning of Day 9, SHR8554 (1 mg) was administered immediately following itraconazole. Blood samples were collected before the infusion (0 h) and at 5 min, the end of infusion, 15 min, 30 min, 45 min, 1 h, 1.5 h, 2 h, 3 h, 4 h, 6 h, 8 h, 12 h, 24 h, 36 h, 48 h after the infusion started on Day 1 and Day 9. Physical examinations, vital signs (blood pressure, heart rate and body temperature), clinical laboratory tests and 12-lead electrocardiogram (ECG) were applied for safety evaluation when necessary.

### Sample size

According to previous study (CTR20180587), which examined SHR8554 blood concentrations obtained through different intravenous infusion times (**Table S2**), the estimated intra-individual variability of AUC was approximately 21.7%. The 90% confidence interval (CI) for the geometric mean ratio (GMR) of AUC comparing combination therapy to monotherapy was set at 80.0 – 125.0%. Assuming a GMR of 100%, a coefficient of variation (CV) of 21.7%, and a tolerance level of 80%, the sample size calculation indicated that with 8 subjects, the 90% CI for the AUC GMR would fall within the predefined range of 80.0–125.0%^24^. Considering that drug interaction clinical trials typically involve 10–20 subjects^25^, and taking into account the self-controlled design used in this trial, the determined sample size for this study was 16 subjects.

### Biological sample analysis

The plasma concentration of SHR8554 were detected using a validated liquid chromatography tandem mass spectrometric (LC-MS/MS) method, mainly involving a Shimadzu 20 A XR ultra-fast liquid chromatography system and a Sciex Triple Quad 6500 + mass spectrometer equipped with a TurboIonSpray^®^ source. The chromatographic separation was conducted in an Agilent Eclipse Plus C18 column (3.5 μm, 50 × 4.6 mm) with the mobile phase consisting of water containing 0.1% formic acid and 10mM ammonium formate (A) and methanol with 0.1% formic acid (B). The flow rate was 0.8 mL/min and the temperature of column oven was set at 40 ℃. The mass spectrometer was operated in positive ion electrospray mode, with quantification obtained using the multiple reaction monitoring (MRM) acquisition mode by monitoring the precursor ion to product ion transitions of m/z 435.3 → 244.3 Da for SHR8554 and 440.3 → 244.3 Da for IS. A low lower limit of quantification (LLOQ) of 0.01 ng/mL was achieved for SHR8554. The precision ranged from 1.50 to 3.30%, and the accuracy ranged from − 1.30 to 1.10% for the linear range of 0.01-10 ng/mL, which was within the acceptable range. Furthermore, the method demonstrated acceptable precision (0.5–3.3%) and accuracy (-2.0 to 3.3%) under various storage conditions. More details of parameters were available in **Supplementary Table S4-S7**.

### Safety assessments

Data on adverse events (AEs), vital signs, physical examinations, clinical laboratory tests and 12-lead ECG were used to tolerability assessments. Medical Dictionary for Regulatory Activities (MedDRA) provides a standardized, internationally recognized terminology for classifying AEs and medical conditions, ensuring consistency in reporting across different regions, regulatory agencies, and pharmaceutical companies. All AEs in this study were coded using the MedDRA (version 24.0) and were graded based on their severity, categorizing them into mild, moderate, or severe. The occurrence rates of AE, drug-related AE and severe adverse events (SAE) were classified based on system organ class (SOC) and preferred term (PT).

### Statistical analysis

For continuous data, summary statistics such as mean and standard deviation were employed. For categorical data, summary statistics included frequency and percentage, offering a comprehensive overview of the distribution of the data. The PK parameters were calculated by using a non - compartment model with Pharsight WinNonlin software (version 8.0, Pharsight, CA, USA). Natural logarithm of main PK parameters (C_max_, AUC_0 − t_, AUC_0−∞_, t_1/2z_, V_z_, CL_z_) was taken and analyzed by linear mixed model. GMR (combination/single dose) and 90% CI were calculated to compare PK characteristics between single and combination administration. Wilconxon paired rank sum test was used to compare T_max_ between the two groups.

## Electronic supplementary material

Below is the link to the electronic supplementary material.

Supplementary Material 1

## Data Availability

The data that support the findings of this study are available from the corresponding author upon reasonable request.
