# Supplementary material for: Exploring Pharmacokinetic interactions between SHR8554, a µ-opioid receptor biased agonist, and Itraconazole in healthy Chinese subjects
Source: Sci Rep. 2025 Jul 2;15:22635. doi: 10.1038/s41598-025-98697-3 (PMC12218957; doi:10.1038/s41598-025-98697-3)
Supplement: Supplementary file 1 — Supplementary Material 1 [file 41598_2025_98697_MOESM1_ESM.docx]

**Supplementary Table S1. Main pharmacokinetic parameters of different doses of SHR8554 in study no.** **CTR20180587.**

|  | **Dose of SHR8554** | | | | |
| --- | --- | --- | --- | --- | --- |
| **Parameters ^a^** | **0.75mg (n=8)** | **1.5mg (n=8)** | **2mg (n=8)** | **2.5mg (n=8)** | **3mg (n=5)** |
| **T_max_ (h) ^b^** | 0.50 (0.33-0.50) | 0.50 (0.33-0.50) | 0.42 (0.33-0.50) | 0.50 (0.33-0.67) | 0.33 (0.17-0.50) |
| **C_max_ (ng/mL)** | 7.41 ± 1.83 | 15.50 ± 3.63 | 17.98 ± 2.99 | 16.93 ± 6.11 | 23.92 ± 6.80 |
| **AUC_0-∞_ (ng.h/mL)** | 16.90 ± 2.44 | 30.01 ± 1.67 | 35.88 ± 1.99 | 45.83 ± 7.05 | 60.30 ± 13.16 |
| **t_1/2_ (h)** | 6.53 ± 1.18 | 6.84 ± 1.39 | 6.08 ± 1.29 | 6.32 ± 1.30 | 6.96 ± 1.69 |
| **CL(L/h)** | 45.12 ± 5.88 | 50.12 ± 2.77 | 55.90 ± 3.10 | 55.77 ± 9.13 | 51.72 ± 11.32 |
| **V(L)** | 424.29 ± 93.44 | 494.75 ± 110.92 | 493.27 ± 124.40 | 501.93 ± 96.94 | 516.73 ± 149.55 |

Note: a. All the subjects received a single intravenous infusion of SHR8554 over 30 min. b. T_max_ is expressed as median (minimum - maximum); other parameters are expressed as Mean ± SD.

**Supplementary Table S2. Main pharmacokinetic parameters of SHR8554 at different infusion times in study no. CTR20180587.**

|  | **Infusion time of SHR8554** | | | |
| --- | --- | --- | --- | --- |
| **Parameters ^a^** | **2 min (n=6)** | **5 min (n=6)** | **15 min (n=6)** | **30 min (n=6)** |
| **T_max_ (h) ^b^** | 0.08 (0.08-0.08) | 0.08 (0.08-0.08) | 0.21 (0.17-0.25) | 0.50 (0.33-0.50) |
| **C_max_ (ng/mL)** | 9.02 ± 3.99 | 15.45 ± 7.67 | 9.04 ± 2.37 | 7.41 ± 1.83 |
| **AUC_0-∞_ (ng.h/mL)** | 12.82 ± 2.05 | 13.94 ± 2.63 | 13.05 ± 2.96 | 16.90 ± 2.44 |
| **t_1/2_ (h)** | 6.68 ± 1.11 | 6.34 ± 1.20 | 6.00 ± 1.29 | 6.53 ± 1.18 |
| **CL(L/h)** | 59.75 ± 9.51 | 55.35 ± 9.79 | 59.94 ± 13.20 | 45.12 ± 5.88 |
| **V(L)** | 576.68 ± 139.44 | 514.08 ± 163.46 | 519.87 ± 168.43 | 424.29 ± 93.44 |

Note: a. All the subjects received a single intravenous infusion of 0.75 mg SHR8554. b. T_max_ is expressed as median (minimum - maximum); other parameters are expressed as Mean ± SD.

**Supplementary Table S3. 90% confidence intervals for major pharmacokinetic parameters of SHR8554 in male and female subjects in the monotherapy and combination therapy groups**

|  | | | **Geometric mean** | | **Geometric mean ratio (M/F)** | |
| --- | --- | --- | --- | --- | --- | --- |
| **Groups** | **Parameters** | **Gender** | **Estimated value** | **95% CI** | **Estimated value** | **90% CI** |
| **Monotherapy**  (**SHR8554**) | C_max_(ng/mL) | Male (n = 11) | 16.30 | (13.91, 19.10) | 1.00 | (0.79, 1.26) |
|  |  | Female (n = 5) | 16.28 | (12.87, 20.61) |  |  |
|  | AUC_0-t_(h*ng/mL) | Male (n = 11) | 16.63 | (14.84, 18.63) | 0.81 | (0.68, 0.95) |
|  |  | Female (n = 5) | 20.58 | (17.39, 24.36) |  |  |
|  | AUC_0-∞_(h*ng/mL) | Male (n = 11) | 16.86 | (15.09, 18.84) | 0.80 | (0.68, 0.95) |
|  |  | Female (n = 5) | 20.95 | (17.77, 24.71) |  |  |
|  | t_1/2z_(h) | Male (n = 11) | 8.06 | (6.31, 10.30) | 0.78 | (0.54, 1.12) |
|  |  | Female (n = 5) | 10.36 | (7.20, 14.89) |  |  |
|  | V_z_(L) | Male (n = 11) | 689.89 | (529.94, 898.13) | 0.97 | (0.66, 1.43) |
|  |  | Female (n = 5) | 713.14 | (482.24, 1054.61) |  |  |
|  | CL_z_(L/h) | Male (n = 11) | 59.30 | (53.07, 66.27) | 1.24 | (1.06, 1.46) |
|  |  | Female (n = 5) | 47.72 | (40.47, 56.27) |  |  |
| **Combination therapy**  **(SHR8554+ itraconazole)** | C_max_(ng/mL) | Male (n = 11) | 14.03 | (9.11, 21.62) | 1.00 | (0.53, 1.88) |
|  |  | Female (n = 5) | 14.07 | (7.41, 26.71) |  |  |
|  | AUC_0-t_(h*ng/mL) | Male (n = 11) | 18.50 | (16.53, 20.69) | 0.87 | (0.74, 1.03) |
|  |  | Female (n = 5) | 21.16 | (17.92, 24.99) |  |  |
|  | AUC_0-∞_(h*ng/mL) | Male (n = 11) | 18.74 | (16.78, 20.93) | 0.86 | (0.73, 1.02) |
|  |  | Female (n = 5) | 21.68 | (18.40, 25.54) |  |  |
|  | t_1/2z_(h) | Male (n = 11) | 7.93 | (6.88, 9.14) | 0.78 | (0.63, 0.96) |
|  |  | Female (n = 5) | 10.19 | (8.25, 12.58) |  |  |
|  | V_z_(L) | Male (n = 11) | 610.45 | (511.77, 728.16) | 0.90 | (0.69, 1.17) |
|  |  | Female (n = 5) | 678.24 | (522.16, 880.97) |  |  |
|  | CL_z_(L/h) | Male (n = 11) | 53.37 | (47.78, 59.61) | 1.16 | (0.98, 1.36) |
|  |  | Female (n = 5) | 46.13 | (39.15, 54.36) |  |  |

**Supplementary Table S4. Calibration curves (n=11).**

| Compound | Standard curves | r | Linear ranges ng/mL | LLOQ Accuracy (RE%) |
| --- | --- | --- | --- | --- |
| SHR8554 | y = 0.256x + 0.000157 | 0.9997 | 0.01-10.0 | 0.0 |

**Supplementary Table S5.** I**ntra-batch and inter-batch precision and accuracy (n=18).**

|  | Nominal concentration(ng/ml) | | Precision (RSD %) | | | | Accuracy (RE %) | | | |  |
| --- | --- | --- | --- | --- | --- | --- | --- | --- | --- | --- | --- |
|  |  |  | intra-day | | inter-day | | intra-day | | inter-day | |  |
| SHR8554 | | 0.03 | | 3.30 | | 3.30 | | 1.10 | | 0.00 | |
|  |  | 1 | | 1.50 | | 2.00 | | -1.27 | | -1.30 | |
|  |  | 8 | | 2.00 | | 2.10 | | 0.56 | | 0.50 | |

**Supplementary Table S6. Extraction recovery and matrix effect data (n=6).**

|  | Nominal concentration(ng/ml) | Recovery (%) | Matrix effect (%) |
| --- | --- | --- | --- |
| SHR8554 | 0.03 | 84.6 | 96 |
|  | 1 | 107.0 | 93 |
|  | 8 | 106.3 | 94 |

**Supplementary Table S7. Stability under different storage conditions (n =6)**

|  | Nominal concentration (ng/ml) | Precision (RSD %) | Accuracy (RE %) |
| --- | --- | --- | --- |
| Short-term  (5 h at 20℃） | 0.03 | 3.2 | 3.3 |
|  | 8 | 1.0 | 1.7 |
| Autosampler  (76 h at 5℃) | 0.03 | 3.3 | 0.0 |
|  | 1 | 1.7 | -1.0 |
|  | 8 | 2.3 | 0.0 |
| Four freeze–thaw cycles  (−70℃ to 20℃） | 0.03 | 3.2 | 3.3 |
|  | 8 | 1.1 | 2.3 |
| Long-term  (731d at −70℃） | 0.03 | 2.4 | 3.3 |
|  | 8 | 0.5 | -2.0 |
